# Stroke in older people in Brazil: prevalence, associated factors, limitations and care practices. A cross-sectional study

**DOI:** 10.1590/1516-3180.2024.0132.R1.13082024

**Published:** 2025-05-26

**Authors:** Priscila Maria Stolses Bergamo Francisco, Athos Paixão Silva Santos, Daniela de Assumpção, Aldiane Gomes de Macedo Bacurau

**Affiliations:** I Department of Collective Health, Faculty of Medical Sciences, Universidade Estadual de Campinas (UNICAMP), Campinas (SP), Brazil.; II Faculty of Medical Sciences, Universidade Estadual de Campinas (UNICAMP), Campinas (SP), Brazil.; III Postgraduate Program in Gerontology, Faculty of Medical Sciences, Universidade Estadual de Campinas (UNICAMP), Campinas (SP), Brazil.; IV Department of Collective Health, Faculty of Medical Sciences, Universidade Estadual de Campinas (UNICAMP), Campinas (SP), Brazil.

**Keywords:** Health services for the aged, Chronic disease, Stroke, Health status disparities, Health Surveys, Factors associated with stroke, Stroke in older people, Guidelines for stroke management, Household survey

## Abstract

**BACKGROUND:**

Cardiovascular diseases, especially ischemic heart disease and stroke, are the main causes of mortality in older people.

**OBJECTIVES:**

To estimate the prevalence of stroke in older people in Brazil, we investigated its associations with sociodemographic factors, health-related behaviors, chronic diseases, body mass index and self-rated health and determined the frequency of limitations related to disease and care practices.

**DESIGN AND SETTING:**

This population-based, cross-sectional study used data from the 2019 Brazilian National Health Survey.

**METHODS:**

This study included older people aged ≥ 60 years (n = 22,728) who answered the question, “Has any doctor ever given you a diagnosis of stroke?” Adjusted odds ratios were estimated using logistic regression.

**RESULTS:**

The prevalence of stroke was 5.6% (95% confidence interval = 5.1-6.1) and was higher in men, individuals aged ≥ 70 years, those with a lower income, those without health insurance, ex-smokers, physically inactive individuals, those with excess weight/obesity, those who rated their own health as fair/poor/very poor and those who reported hypertension, diabetes, kidney failure, depression and heart disease. The most adopted care practices were follow-ups by a healthcare provider (60.8%), medication use (59.3%) and diet (47.2%). Only 17.0% of patients underwent physiotherapy, and 53.7% reported limitations in habitual activities.

**CONCLUSIONS:**

Subgroups of people more affected by stroke were identified, which included older individuals, those with lower socioeconomic status, ex-smokers and those with neuroendocrine and cardiovascular comorbidities. Less than 20% of patients underwent physiotherapy, which underscores the need to expand multidisciplinary care in the health network.

## INTRODUCTION

Cardiovascular diseases (CVDs) constitute the primary cause of death worldwide, with stroke ranking as the second leading cause of death and the third leading cause of death and disability combined.^1^ Each year, approximately 15 million individuals worldwide experience a stroke, resulting in five million deaths, while another five million individuals develop permanent disabilities, imposing a burden on their families and communities.^2^ Furthermore, approximately 90% of stroke survivors reside in lower-income and lower-middle-income countries.^1,3^

In Brazil, 1.5% of the population aged ≥ 18 years had a diagnosis of stroke in 2013 (approximately 2.2 million) and the prevalence was 5% among older people (aged ≥ 60 years).^4^ The prevalence of stroke in the population aged ≥ 18 years was 2.0% in 2019 (3.1 million) and increased with age.^5^ Additionally, an increase was observed in comorbidities associated with stroke, such as physical, hearing and intellectual impairments, among others that were not always considered to result from stroke but could exert a negative impact on quality of life.^6^ Stroke is one of the main causes of disability, contributing significantly to disability-adjusted life years.^1,2,7^

The sociodemographic factors associated with stroke include age, sex, ethnicity/race, education, socioeconomic status and the region of origin.^1,3,8-10^ Moreover, smoking, a sedentary lifestyle and alcohol abuse are behavioral factors that increase the risk of stroke.^3,6,11-13^ High blood pressure and smoking are the most common modifiable risk factors for stroke.^1,2^ Indeed, four out of every 10 deaths could have been avoided if the blood pressure of the individuals had been controlled.^2,12^ Additionally, healthy dietary habits are important for the treatment and control of hypertension and type 2 diabetes, which are risk factors for CVDs, including stroke.^13^

Stroke is an important cause of morbidity and mortality in Brazil, and modifiable risk factors account for nearly half of the deaths due to the disease.^2^ An unhealthy lifestyle, together with age and chronic diseases, contributes significantly to the increase in the occurrence of stroke, which has physical, psychological and financial impacts on affected individuals, as well as their families, healthcare services and society, especially in regions with lower socioeconomic development.^7^ Estimates of the magnitude of stroke in older people and specific subgroups of the population are scarce in the literature and can contribute to the prevention of risk factors, the expansion of treatment, the control of comorbidities and more comprehensive care in the primary care network.

## OBJECTIVE

To estimate the prevalence of stroke among older people in Brazil, identify the associated sociodemographic factors, health-related behaviors, chronic diseases, body mass index and self-rated health and determine the frequency of care practices.

## METHODS

This population-based cross-sectional study was conducted with data from older people (aged ≥ 60 years; n = 22,728) who participated in the 2019 Brazilian National Health Survey,^14^ considering the greater occurrence of stroke in the older population. Public domain data are available at https://www.pns.icict.fiocruz.br/bases-de-dados/.

The survey used cluster sampling in three stages. The primary sampling units were randomly selected and comprised census sectors/sets of sectors. Permanent private homes were selected from each primary sampling unit using simple random sampling. Finally, residents were selected from each home using simple random sampling to answer the questionnaire. Details of the sampling process, weighting factors and content of the data collection instrument have been published elsewhere.^14^

Herein, data were collected from all older people who answered the question *Has any doctor ever given you a diagnosis of stroke*? (yes/no). Among those who answered “yes”, the following question was posed: *How old were you when you received your first diagnosis of stroke*? (in years) *What do you currently do because of the stroke*? (dieting, physical therapy, other rehabilitation therapies, take aspirin regularly, take other medications or undergo regular monitoring by a healthcare provider; each with ‘yes’ and ‘no’ response options); *In general, to what degree does the stroke limit your habitual activities, such as work, doing household chores, etc.*? (not at all, a little, moderately, intensely or very intensely).

The following variables were considered in the analysis of sociodemographic factors, behaviors and comorbidities:

- Sociodemographic: sex (male; female), age group (60-69; 70-79; ≥ 80 years), race/skin color (white/yellow; black/brown/indigenous), schooling (no schooling/incomplete primary school education; complete primary school/complete high school education; incomplete/complete university/college education), family income per capita using the monthly minimum wage (MMW) as reference (up to 1/2 the MMW; > 1/2 up to MMW; > MMW up to two times the MMW; > two times up to three times the MMW; more than three times the MMW) and whether the respondent had health insurance (yes/no).
- Health-related behaviors: smoking (non-smoker; current smoker; ex-smoker), habitual consumption of alcoholic beverages (< once per month; ≥ once per month), practice of physical activity (PA) during leisure (active: at least 150 minutes/week of light-to-moderate PA or 75 minutes of vigorous PA; inactive/insufficiently active: ≤ 150 minutes/week) and food intake indicator (healthy/unhealthy) determined from questions on the weekly consumption of beans, vegetables, fruit and milk (markers of healthy dietary patterns) and red meat, sweetened beverages and sweets (markers of unhealthy patterns). Depending on the food and intake frequency, the scores ranged from zero to four; the minimum score was attributed to healthy foods consumed daily and unhealthy foods never or hardly ever consumed. The maximum score was attributed when healthy foods were consumed at a frequency of ‘never/hardly ever’ and unhealthy foods were consumed every day of the week. This indicator ranged from 0 to 28 points, with higher scores indicating worse dietary patterns. The total score was classified in terciles (2^nd^ and 3^rd^ terciles were grouped) to generate an unhealthy diet variable (≥ 8 points).
- Body mass index (BMI): interpreted according to the cutoff points recommended by the Pan American Health Organization: BMI < 23 (underweight), BMI ≥ 23 and < 28 (ideal range), BMI ≥ 28 and < 30 (overweight) and BMI ≥ 30 (obesity).^15^ Overweight and obesity were categorized as excess weight.
- Self-reported chronic diseases and self-rated health: hypertension, diabetes mellitus, heart diseases (myocardial infarction, angina, heart failure or others), high cholesterol, lung disease or chronic obstructive pulmonary disease (COPD), asthma or asthmatic bronchitis, arthritis or rheumatism, kidney failure, cancer and depression (yes/no); and self-rated health (very good/good; fair; poor/very poor).

Descriptive analyses were performed to characterize the study population. The rates of stroke and 95% confidence intervals (CI) were estimated according to sociodemographic variables, health behaviors and chronic diseases/health conditions. Associations between stroke and the variables of interest were assessed using Pearson’s chi-square test (Rao & Scott) with a 5% significance level. Odds ratios (ORs) adjusted for sex and age were estimated using logistic regression to express the magnitude of the associations.

A hierarchical multiple regression model was then applied considering three blocks of variables: sociodemographic variables with a p-value < 0.20 were incorporated first. Next, variables that maintained significance after adjusting for other variables at the same level were retained, and behaviors and BMI were incorporated. After adjusting for this set of variables, those that maintained statistical significance were retained, and chronic diseases and self-rated health were incorporated. All variables with a P value < 0.05 after adjusting for variables at the higher level and the same hierarchical level were retained in the final model.

The analyses were performed using the *survey* module of Stata 15, with weights inherent to the complex sampling design of the study. The 2019 National Health Survey was approved by the National Research Ethics Committee (process nº3.529.376; August 23^rd^, 2019). All interviewees received clarifications regarding the objectives of the study and agreed to participate.^14^

## RESULTS

The mean age of included older people was 69.9 years (95%CI = 69.7-70.0) and most were women (56.7%), of white/yellow race/skin color (51.8%), had a spouse (56.3%), had no schooling/incomplete primary school education (63.3%), had an income less than the MMW (41.7%) and did not have health insurance (70.7%). The prevalence of stroke was 5.6% (95%CI = 5.1-6.1) and the age at the first diagnosis was ≥ 60 years in 65.2% (95%CI = 61.1-69.1) of patients. The prevalence of stroke was higher among men, individuals aged ≥ 70 years, black/brown individuals, those with lower schooling, those with a lower income and those who did not have health insurance (Table 1).

**Table 1:** Percentage distribution and prevalence of stroke in older people (aged ≥ 60 years) according to sociodemographic characteristics. 2019 National Health Survey

Variables/categories	n (%)	% (95%CI)	P value
*Sex*			
Male	10,193 (43.32)	6.40 (5.64-7.25)	**0.0052**
Female	12,535 (56.68)	4.99 (4.44-5.62)	
*Age group (in years)*			
60 to 69	12,555 (56.30)	3.79 (3.32-4.33)	**< 0.0001**
70 to 79	7,157 (30.14)	7.68 (6.66-8.83)	
80 and more	3,016 (13.56)	8.50 (6.99-10.30)	
*Race/skin color* ^1^			
White/Yellow	10,105 (51.79)	4.88 (4.29-5.55)	**0.0025**
Black/Brown/Indigenous	12,621 (48.21)	6.37 (5.68-7.15)	
*Marital status*			
With spouse	10,725 (56.27)	5.33 (4.72-6.01)	0.2100
Without spouse	12,003 (43.73)	5.95 (5.26-6.73)	
*Schooling*			
None/incomplete primary school	14,987 (63.27)	6.55 (5.94-7.23)	**< 0.0001**
Complete primary/complete high school	5,040 (24.03)	4.40 (3.62-5.34)	
Incomplete/complete university/college	2,701 (12.70)	3.13 (2.37-4.12)	
*Income per capita (MMW)*			
≤ 1/2	2,393 (10.19)	7.53 (5.78-9.75)	**< 0.0001**
> 1/2 to ≤ 1	7,857 (31.54)	6.72 (5.83-7.73)	
>1 to ≤ 2	6,617 (31.87)	5.41 (4.62-6.32)	
> 2 to ≤ 3	2,287 (10.80)	4.35 (3.27-5.77)	
> 3	3,571 (15.60)	3.35 (2.60-4.32)	
*Has health insurance*			
Yes	5,837 (29.28)	6.28 (5.69-6.92)	**< 0.0001**
No	16,891 (70.72)	3.96 (3.30-4.74)	

n (%) = absolute number of observations (percentage of weighted sample); CI = confidence interval; P value of chi-square test (Rao-Scott); MMW = monthly minimum wage.

A greater likelihood of stroke was found among ex-smokers versus non-smokers (OR = 1.3; 95%CI = 1.1-1.6), those with excess weight compared with those in the ideal weight range (OR = 1.4; 95%CI = 1.1-1.7) and inactive/insufficiently active individuals (OR = 1.6; 95%CI = 1.2-2.1) after adjusting for sex and age. The prevalence was lower among those who reported alcohol consumption at least once per month (Table 2).

**Table 2:** Prevalence of stroke in older people (aged = 60 years) and odds ratios according to health-related behaviors and body mass index. 2019 National Health Survey

Variables/categories	%	95%CI	P value	OR_adjusted_(95%CI)
*Smoking*				
Non-smoker	4.95	4.35-5.63	**0.0063**	1
Smoker	4.69	3.44-6.36		1.05 (0.74-1.49)
Ex-smoker	6.56	5.78-7.45		**1.29 (1.06-1.57)**
*Habitual frequency of alcohol intake*				
Never or < once per month	6.28	5.74-6.87	**< 0.0001**	1
≥ once per month	2.68	2.09-3.43		**0.42 (0.31-0.55)**
*Practice of physical activity during leisure*				
Active	3.53	2.77-4.50	**< 0.0001**	1
Insufficiently active/inactive	6.11	5.59-6.68		**1.58 (1.22-2.06)**
*Dietary intake*				
Healthy	6.09	5.34-6.93	0.0867	1
Unhealthy	5.25	4.69-5.87		0.87 (0.73-1.05)
*Body mass index*				
Underweight	6.10	5.03-7.37	0.0756	1.14 (0.90-1.46)
Ideal range	4.98	4.38-5.65		1
Overweight/obesity	6.13	5.30-7.08		**1.37 (1.12-1.68)**

CI = confidence interval; P-value of chi-square test (Rao-Scott); OR_adjusted_ = odds ratio adjusted for sex and age.

The prevalence of stroke was higher among individuals who reported chronic diseases, except for asthma and cancer. The prevalence of stroke was 15.1% (95%CI = 13.1-17.4) among individuals who self-rated their health as poor/very poor; this association remained strong after adjustments for sex and age (OR = 5.8; 95%CI = 4.5-7.5) (Table 3). All variables independently associated with stroke are presented in Table 4.

**Table 3:** Prevalence of stroke in older people (aged = 60 years) and odds ratios according to chronic disease and self-rated health. 2019 National Health Survey

Variables/categories	%	95%CI	P value	OR_adjusted_(95%CI)
*Arterial hypertension*				
No	3.27	2.69-3.98	**< 0.0001**	1
Yes	7.46	6.79-8.19		**2.34 (1.86-2.93)**
*Diabetes mellitus*				
No	4.86	4.37-5.39	**< 0.0001**	1
Yes	8.62	7.38-10.04		**1.85 (1.51-2.26)**
*Heart disease*				
No	4.42	3.97-4.93	**< 0.0001**	1
Yes	13.40	11.59-15.45		**3.07 (2.49-3.78)**
*High cholesterol*				
No	4.96	4.45-5.53	**< 0.0001**	1
Yes	7.47	6.49-8.59		**1.76 (1.45-2.14)**
*Lung disease/COPD*				
No	5.43	4.97-5.92	**< 0.0001**	1
Yes	11.46	8.13-15.92		**1.99 (1.36-2.91)**
*Asthma/asthmatic bronchitis*				
No	5.52	5.05-6.03	0.1304	1
Yes	7.21	5.16-10.0		1.35 (0.93-1.96)
*Arthritis/rheumatism*				
No	5.42	4.92-5.97	0.1194	1
Yes	6.42	5.32-772		**1.28 (1.02-1.60)**
*Kidney failure*				
No	5.38	4.92-5.89	**< 0.0001**	1
Yes	13.93	10.24-18.66		**2.67 (1.85-3.84)**
*Cancer*				
No	5.44	4.98-5.95	**0.0224**	1
Yes	7.76	5.80-10.32		1.31 (0.94-1.83)
*Depression*				
No	5.06	4.60-5.57	**< 0.0001**	1
Yes	9.62	7.92-11.65		**2.34 (1.85-2.96)**
*Self-rated health*				
Very good/good	2.91	2.42-3.49	**< 0.0001**	1
Fair	6.08	5.36-6.88		**2.08 (1.66-2.62)**
Poor/very poor	15.12	13.09-17.41		**5.77 (4.46-7.46)**

CI = confidence interval; P value of chi-square test (Rao-Scott); OR_adjusted_ = odds ratio adjusted for sex and age.

**Table 4:** Hierarchical multiple logistic regression model for associations between stroke and sociodemographic variables, health-related behaviors and health conditions in older people (aged = 60 years). 2019 National Health Survey

Variables/categories	First step OR^a^_adjusted_ (95%CI)	Second step OR^b^_adjusted_ (95%CI)	Third step OR^c^_adjusted_ (95%CI)
*Sex*			
Female	1		
Male	1.33 (1.10-1.60)		
*Age group*			
60 – 69	1		
70 – 79	2.13 (1.73-2.63)		
80 and more	2.42 (1.87-3.15)		
*Income per capita (MMW*)			
≤ 1/2	1		
> 1/2 to ≤ 1	0.78 (0.56-1.08)		
> 1 to ≤ 2	0.67 (0.48-0.94)		
> 2 to ≤ 3	0.57 (0.37-0.88)		
> 3	0.48 (0.31-0.73)		
*Has health insurance*			
Yes	1		
No	1.31 (1.01-1.71)		
*Smoking*			
Non-smoker		1	
Smoker		1.06 (0.74-1.53)	
Ex-smoker		1.33 (1.10-1.62)	
*Frequency of alcohol intake*			
Never or < once per month		1	
≥ once per month		0.45 (0.34-0.61)	
*Practice of physical activity during leisure*			
Active		1	
Insufficiently active/inactive		1.43 (1.09-1.88)	
*Dietary intake*			
Healthy		1	
Unhealthy		0.82 (0.68-0.98)	
*Body mass index*			
Underweight		1	
Ideal range		1.10 (0.86-1.40)	
Overweight/Obesity		1.33 (1.08-1.63)	
*Chronic diseases*			
Arterial hypertension			1.78 (1.39-2.28)
Diabetes mellitus			1.27 (1.02-1.59)
Heart disease			2.15 (1.74-2.67)
Kidney failure			1.62 (1.10-2.37)
Depression			1.81 (1.41-2.33)
*Self-rated health*			
Very good/good			1
Fair			1.48 (1.15-1.91)
Poor/very poor			3.22 (2.40-4.32)

MMW = monthly minimum wage; CI = confidence interval; OR = odds ratio.

^a^adjustment for sociodemographic characteristics;

^b^adjustment for sociodemographic characteristics, health-related behaviors and body mass index;

^c^adjustment for sociodemographic characteristics, health-related behaviors, body mass index and variables at the same hierarchical level.

**Figure 1:**
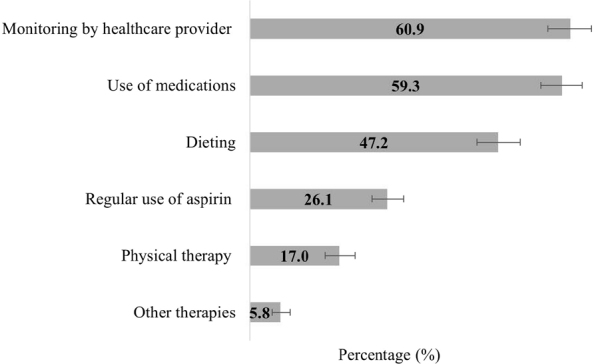
Percentage distribution of care practices performed by older people (aged ≥ 60 years) because of stroke. 2019 National Health Survey.

Regarding care practices, physical therapy was reported by 17% of older adults and dieting was reported by 47%. Approximately 60% reported undergoing regular monitoring by a healthcare provider, 26.1% took aspirin and more than 50% reported taking other medications because of stroke. Moreover, approximately 59% reported limitations in the performance of habitual activities (Figure 1).

## DISCUSSION

The results showed that the prevalence of stroke differed according to sociodemographic characteristics, health behaviors and comorbidities. Dieting was the most frequent care practice reported by stroke survivors. More than 50% of the patients used medications other than aspirin for the disease, underwent regular monitoring by a healthcare provider and reported some limitations in the performance of activities of daily living.

Studies with adults and older people report differences in the prevalence of stroke between sexes.^1,7-10^ Some report a greater occurrence of the disease among men, with this difference tending to diminish with age. Women have their first stroke approximately 4 years later than men, and although ischemic or hemorrhagic stroke is more frequent in men, cardioembolic stroke, which tends to be more lethal, is more frequent in women.^10^ The differences between the sexes may partly be explained by differences in risk factors, such as the control of hypertension, BMI, dyslipidemia, alcohol use, smoking and medicinal treatment.^10,13^ Smoking is an independent risk factor for the occurrence of stroke in both sexes but the habit is more frequent among men,^13^ especially because of social, cultural and economic issues.

Furthermore, sex differences regarding healthcare and prevalence of diseases are found among older people.^10^ The late diagnosis of diseases, such as hypertension, is more frequent among men. The Longitudinal Study on the Health of Older Brazilians found that approximately 20% of the population aged ≥ 50 years did not have a diagnosis of hypertension and alcohol intake, especially among men, increased the likelihood of the non-diagnosis of the disease.^16^

Age is associated with stroke, and the absolute number of individuals with the disease tends to increase with the aging of the population.^1-3,12,17^ Moreover, an increase has been observed in the mortality of older people.^10^ As aging *per se* predisposes the organism to chronic diseases, the risk of stroke doubles for each successive decade over the age of 55 years.^12^ Herein, the prevalences of stroke were 0.3%, 1.7% and 5.6% among individuals aged 20 to 39, 50 to 59 and ≥ 60 years, respectively, demonstrating the influence of age on the occurrence of the disease. Over time, functional domains undergo physiological decline due to cellular aging and degenerative processes (such as the stiffening of blood vessels), which can result in various diseases and are influenced by genetic, behavioral and environmental factors.^18^

In the bivariate analysis, the prevalence of stroke was higher among older adults with a lower level of schooling. Indeed, low schooling is significantly associated with a greater risk of stroke.^1,8,9,11^ Besides being an important determinant of socioeconomic status, schooling level exerts a strong influence on health-related practices, attitudes and behaviors, as it is also a factor that influences seeking and using healthcare services. Higher levels of schooling are related to higher income and better living standards and may influence health standards.^15^

Social inequalities in the occurrence of stroke are determined by the indicators of income and health insurance, as less access to healthcare services limits opportunities for counseling on the prevention of complications associated with chronic diseases, which are generally found in older people.^19^ Moreover, socioeconomic differences are reflected in differences related to mortality due to CVDs, especially stroke.^1^

The prevalence of stroke was higher among ex-smokers, demonstrating the harmful effects of tobacco on cerebrovascular outcomes and underscoring the importance of encouraging and supporting the management of modifiable risk factors and lifestyle changes.^20,11,13^ Exposure to toxic substances due to smoking activates several mechanisms that predispose smokers to the occurrence of cerebrovascular diseases and worse outcomes after stroke.^12,20^ One study identified a trend toward an increase in the prevalence of ex-smokers in Brazil.^21^ Thus, strategies aimed at reducing smoking and promoting a healthy lifestyle may reduce the burden of stroke.^2,12^ Multidisciplinary therapeutic groups in primary care services can play an important role beyond the conventional therapy offered.^22^

The lower incidence of stroke among participants who reported consuming alcoholic beverages should be interpreted considering the limitations inherent in the cross-sectional design of this study. This finding could result from survival bias when obtaining information from older people who have survived risk exposure throughout life. However, a previous study that investigated alcohol intake patterns in older Brazilians found that stroke was associated with mild-to-moderate alcohol intake.^23^

A higher prevalence of stroke has been observed among inactive and insufficiently active older adults. Behavioral factors, such as smoking, low physical activity and poor diet, account for 47.4% of stroke burden.^3^ The benefits of physical activity for health are widely described in the literature for the prevention of chronic diseases and their complications.^24^ One study identified that physical inactivity is a treatable risk factor for stroke even among individuals aged > 80 years and suggested that improving activity may reduce the risk of stroke in this segment of the population.^25^

From 1990 to 2019, dietary risks (diet high in sodium and red meat, diet low in fruits, vegetables, whole grains and alcohol consumption) accounted for 30.0% of stroke-related disability-adjusted life years.^3^ Herein, the prevalence of stroke was lower among participants with unhealthy dietary patterns. This finding is likely the result of reverse causality, which is an inherent disadvantage of a cross-sectional design, as exposure and outcome are measured at a single moment in time. Sequelae of stroke, such as the loss of sensory function, cognitive function, functional capacity, communication and emotional problems, exert a negative impact on food intake.^26,27^ Among stroke survivors, approximately 40% have persistent dysphagia, which in turn increases the risk of pneumonia (especially in cases of silent aspiration), malnutrition and dehydration.^26,27^

The prevalence of stroke was higher in participants with excess weight. A meta-analysis of 20 studies identified a greater risk of stroke among individuals classified as overweight and with obesity compared with those in the ideal weight range, and the risk of ischemic stroke was 53% and 68% higher in these two excess weight groups, respectively.^28^ Explanatory mechanisms of the effect of obesity to brain vessels include associations with hypertension, diabetes and hyperlipidemia, which are chronic diseases that promote the development of arteriosclerosis and the production of adipokines, such as leptin and tumor necrosis factor, which cause inflammation and atherosclerosis.^28^ Although the risk of stroke related to BMI, low-density lipoprotein cholesterol, systolic arterial pressure, glycemia or smoking diminishes with age, these risk factors are generally present concurrently in older people, significantly modifying the importance of such factors in the occurrence of stroke.^17^

The focus should be maintained on risk factors, as well as the presence of comorbidities.^12,17^ For instance, studies have demonstrated that COPD may be a risk factor for stroke; however, as individuals can have multiple comorbidities, it is difficult to state whether COPD is an independent risk factor for stroke or whether the association is due to a confounding effect,^29^ which can be applied to the older population, considering the diseases investigated and the cross-sectional nature of the study. The prevalence of certain conditions that increase the probability of stroke, such as diabetes, hypertension, atrial fibrillation and coronary and peripheral arterial diseases, increases with age and affects older people more.^17^

Morbidities, whether alone or combined, potentially interact with conventional cardiovascular risk factors to modify the risk of stroke in older people.^17^ Although no association was found between cancer and stroke herein, patients with cancer have an increased risk of arterial thrombosis, which increases the possibility of myocardial infarction and stroke.^30^ Treatment and lifestyle interventions directed at other morbidities, such as diseases of the circulatory system, diabetes, cancer, arrhythmia and heart failure, can contribute to a reduction in the occurrence of stroke in this subgroup.^31^

The present results indicate the impact of stroke and the limitations imposed by the disease on self-rated health and quality of life, as 72% of the participants who reported some limitations due to the disease considered their health to be poor or very poor. Self-rated health is an indicator of actual health status, including among older people,^32^ as those who report poor health have been demonstrated to be consistently at a greater risk of mortality than those who report better health.^15^

The use of medications by older people who have had a stroke may also reflect their relationship with other diseases/chronic conditions. Although the drugs taken were not the object of investigation, good medication prescription practices^13^ must be observed when providing healthcare for older people at primary care services to avoid iatrogenesis associated with prescriptions, as side effects related to medications are more common in the older population^33^ and can be confounded by common symptoms such as fatigue caused by beta-blockers, which can lead to depression.^33^

Regarding care practices, health problems that impose physical limitations, mobility decline and/or cognitive impairment, such as stroke, can impede the execution of habitual physical activities. In the present study, only 17% of participants who reported a diagnosis of stroke underwent physical therapy, despite the fact that such treatment can rehabilitate individuals and improve their performance of physical activities and mobility, which can exert a positive impact on health and quality of life.^6^

Access to care is fundamental to the maintenance of the health of older people with stroke, and the intensity and frequency of rehabilitation correlate with functional improvement.^6,13^ Regarding post-stroke rehabilitation, its integration within the entire health system and social community care is needed.^26,34^ Physical therapy is essential to the recovery and improvement of mobility through the rehabilitation of gait, functional recovery and social (re)inclusion.^6^ The program should begin with primary care services through a multi-professional team, as early diagnosis of limiting/disabling conditions enables immediate intervention and the development of an interdisciplinary care plan.^6^

Many older people who survive a stroke experience sequelae that make them dependent on others for activities of daily living, which significantly impacts individuals, family members and caregivers. Stroke rehabilitation requires a coordinated, sustained effort by a multiprofessional team, including the patient, family, caregiver and healthcare providers, with coordination and effective communication among all involved.^27^

A healthy, varied diet is essential for the non-pharmacological treatment of the disease.^6,11-13^ Herein, dieting was reported by 47% of stroke survivors, underscoring the importance of providing information on the benefits of a healthy diet and expanding the actions of integral care offered by the Brazilian public health system, such as home care for individuals with stroke-related sequelae and the strengthening of multidisciplinary teams. To promote cardiovascular health, individuals should consume a large variety of fruits and vegetables, whole grains, plant-based sources of protein (beans and seeds/nuts), fish, low-fat dairy products and vegetable oils and avoid sweetened beverages, sweets, processed meats and salt to promote cardiovascular health.^13,35^

The strengths of present study include the use of representative data for community-dwelling older Brazilians, enabling the delineation of the epidemiological profile of stroke in this population with the aim of reducing the impact of the disease, promoting quality of life and diminishing the burden of the disease on healthcare services.^34^ Among the limitations of the study, the cross-sectional design does not enable establishing temporal relationships between variables, and some associations may have been due to reverse causality. Information bias is also a possibility, as self-reported medical diagnoses of chronic diseases may be subject to recall bias and access to healthcare services.

## CONCLUSION

Inverse associations were found between the prevalence of stroke and socioeconomic level of the older people analyzed (reflected by income and access to health insurance). The occurrence of stroke was greater among ex-smokers and individuals with neuroendocrine and cardiovascular comorbidities. Less than 20% of individuals with the disease reported undergoing physical therapy, revealing the need to expand multi-professional care in the healthcare network for this subgroup. These findings contribute to the identification of segments of the older population that are more affected by stroke and could assist in the planning of future interventions.
